# Inequalities in the Use of eHealth Between Socioeconomic Groups Among Patients With Type 1 and Type 2 Diabetes: Cross-Sectional Study

**DOI:** 10.2196/13615

**Published:** 2019-05-29

**Authors:** Anne Helen Hansen, Meghan Bradway, Jan Broz, Tor Claudi, Øystein Henriksen, Silje C Wangberg, Eirik Årsand

**Affiliations:** 1 Centre for Quality Improvement and Development University Hospital of North Norway Tromsø Norway; 2 Department of Community Medicine Faculty of Health Sciences UiT–The Arctic University of Norway Tromsø Norway; 3 Norwegian Centre for eHealth Research University Hospital of North Norway Tromsø Norway; 4 Department of Clinical Medicine University Hospital of North Norway Tromsø Norway; 5 Department of Internal Medicine Second Faculty of Medicine Charles University Prague Czech Republic; 6 Department of Medicine Nordland Hospital Bodø Norway; 7 Faculty of Social Sciences Nord University Bodø Norway; 8 Department of Health and Care Sciences Faculty of Health Sciences UiT–The Arctic University of Norway Narvik Norway; 9 Department of Clinical Medicine UiT–The Arctic University of Norway Tromsø Norway

**Keywords:** inequalities, eHealth, internet, health care utilization, cross-sectional study, diabetes mellitus, type 1, diabetes mellitus, type 2, education, income, Norway

## Abstract

**Background:**

The prevalence of diabetes and the use of electronic health (eHealth) are increasing. People with diabetes need frequent monitoring and follow-up of health parameters, and eHealth services can be highly valuable. However, little is known about the use of eHealth in different socioeconomic groups among people with diabetes.

**Objective:**

The aim of this study was to investigate the use of 4 different eHealth platforms (apps, search engines, video services, and social media sites) and the association with socioeconomic status (SES) among people diagnosed with type 1 and type 2 diabetes mellitus (T1D and T2D, respectively).

**Methods:**

We used email survey data from 1250 members of the Norwegian Diabetes Association (aged 18-89 years), collected in 2018. Eligible for analyses were the 1063 respondents having T1D (n=523) and T2D (n=545). 5 respondents reported having both diabetes types and thus entered into both groups. Using descriptive statistics, we estimated the use of the different types of eHealth. By logistic regressions, we studied the associations between the use of these types of eHealth and SES (education and household income), adjusted for gender, age, and self-rated health.

**Results:**

We found that 87.0% (447/514) of people with T1D and 77.7% (421/542) of people with T2D had used 1 or more forms of eHealth sometimes or often during the previous year. The proportion of people using search engines was the largest in both diagnostic groups, followed by apps, social media, and video services. We found a strong association between a high level of education and the use of search engines, whereas there were no educational differences for the use of apps, social media, or video services. In both diagnostic groups, high income was associated with the use of apps. In people with T1D, lower income was associated with the use of video services.

**Conclusions:**

This paper indicates a digital divide among people with diabetes in Norway, with consequences that may contribute to sustaining and shaping inequalities in health outcomes. The strong relationship between higher education and the use of search engines, along with the finding that the use of apps, social media, and video services was not associated with education, indicates that adequate communication strategies for audiences with varying education levels should be a focus in future efforts to reduce inequalities in health outcomes.

## Introduction

### Increasing Prevalence of Diabetes

The prevalence of diabetes is increasing worldwide; it is expected to rise to 642 million cases in 2040 [1]. Global prevalence in adults is estimated at 8.8% [1]. Around 245,000 people have been diagnosed with diabetes in Norway, of whom around 28,000 (11.4%) have type 1 diabetes (T1D) [2]. Most patients do not reach the combined national treatment targets for prevention of complications [3-5].

### Increasing Use of Electronic Health

The World Health Organization defines electronic health (eHealth) as “the use of information and communication technologies for health” [6]. The use of eHealth has increased significantly over the past decades, and around 80% of the general population in the United States and Europe conducts health-related searches on the Web [7-10]. It has recently been reported that 87% of people with T1D in Norway used eHealth in 1 or more forms, and 84% of people with T1D had used search engines sometimes or often during the previous year [11]. Most Norwegian households (98%) have internet access [12], 96% of the population aged 16 to 79 years has used the internet during the previous 3 months, and 90% of this population uses the internet every day [13]. Social media is used by 80% of the Norwegian population [13].

### Socioeconomic Inequalities in the Prevalence, Morbidity, and Mortality of Diabetes

Despite a relatively high average standard of living, all European countries still have substantial inequalities in health outcomes among socioeconomic groups, as affluent groups have better somatic and mental health and lower mortality than disadvantaged groups [14]. Relative health differences between the highest and lowest socioeconomic groups in Norway even rank among the highest in Europe [14]. The inverse association between socioeconomic status (SES) and the prevalence, morbidity, and mortality of diabetes is well documented [15,16]. In addition, there is evidence of worse health care for diabetes patients with low SES [15,17]. Health care services and individuals’ abilities to take advantage of them are considered parts of the explanations for inequalities in health, even in universal health care systems [17,18].

### Socioeconomic Inequalities in the Use of Electronic Health

One might assume that communication inequalities can contribute to inequalities in health, as it is well known that new interventions and treatments reach people in higher socioeconomic groups first [19-21]. Research consistently indicates that women, younger people, and people with middle and high SES are more likely to seek health information and advice from the internet [22-25]. In addition, both long-term illness and good health are reported to be positively associated with eHealth use [8,22]. However, in the case of diabetes, there is evidence that there is no immediate benefit from health technology implementation in lower-education groups, in contrast to medium- and especially higher-education groups, with possible consequences regarding health outcomes [24]. Wangberg et al found that SES is related to differential use of eHealth, as people with higher education use eHealth tools that more likely influence health behaviors [26]. Given access to the internet, the digital health divide implies that some people are less likely to use the internet for health purposes, as well as to benefit from eHealth resources [10,25].

Despite recent reports of a decrease in gender and health disparities in the use of eHealth, persistent predictors of less use of eHealth seem to be higher age (75 years and older), lower education (lower than high school), and (very) low income [9].

### Diabetes and Electronic Health

Type 2 diabetes (T2D) is partly caused, maintained, and deteriorated by preventable risk factors, such as physical inactivity, unhealthy diet, obesity, and smoking. In daily life, individuals with diabetes are in charge of managing their disease and self-management, and empowerment is essential in care and prevention of complications of T1D and T2D diabetes. Recent systematic reviews have shown that eHealth can play a positive role in this regard [27-29]. It is a core political ambition to equalize social inequalities in health, as reflected in the Norwegian Public Health Act [30]. To our knowledge, no one has studied the relationship among SES groups in the form of education and household income, and different forms of eHealth used by people with diabetes in Norway. As there is consistent evidence of socioeconomic inequalities in health, as well as inequalities in the distribution of diabetes and in the use of eHealth, studies of possible gaps in information seeking among people with diabetes deserve close attention. This is equally relevant in relation to the importance of information in the follow-up of this prevalent and lifelong chronic disease.

### Objective

The aim of this study was to investigate the use of different eHealth platforms among people with diabetes (T1D and T2D) and investigate whether the use of eHealth was associated with SES. Specifically, we tested whether the use of apps, search engines (such as Google), video services (such as YouTube), and social media (such as Facebook) was associated with education and household income, adjusted for gender, age, and self-rated health.

## Methods

### Data

The current cross-sectional study is a part of the DIAcare project [31], investigating relations between eHealth use and the use of provider-based health care services. The project has previously published 3 papers, partly using the same dataset and methodology [11,32,33]. Initially, as described in our protocol paper [31], we planned to use data from the seventh Tromsø Study, conducted in 2015-2016. However, these data were not available to us because of an agreement with another researcher regarding exclusive rights to decide about the collected eHealth data for 3 years. Consequently, we developed a tailored questionnaire on the basis of the specific objectives of this study [31], using relevant questions from other published surveys on health seeking behavior [34,35].

Email survey data were obtained in January and February 2018 from members of The Norwegian Diabetes Association (NDA). By December 31, 2017, the patient organization had 33,908 members, 53% were women and 47% were men. Around 30% of the members have T1D [36]. The Norwegian Centre for Research Data (NSD) Web Survey distributed the invitations to a randomly selected sample of 5971 individuals (about 18% of all members). We distributed information about the study purpose and what participation would entail together with the invitation. The questionnaire (Multimedia Appendix 1) included questions about demographic and socioeconomic characteristics, health status, including specific questions about duration, severity and treatment of the individuals’ diabetes, and use of and experiences with eHealth. Before data collection, 2 people diagnosed with diabetes and 2 experts from our research group (EÅ and AHH) reviewed and tested the questionnaire several times. Nonrespondents were given 1 reminder, submitted by email 15 days after the first request.

### Participants

**Figure 1:**
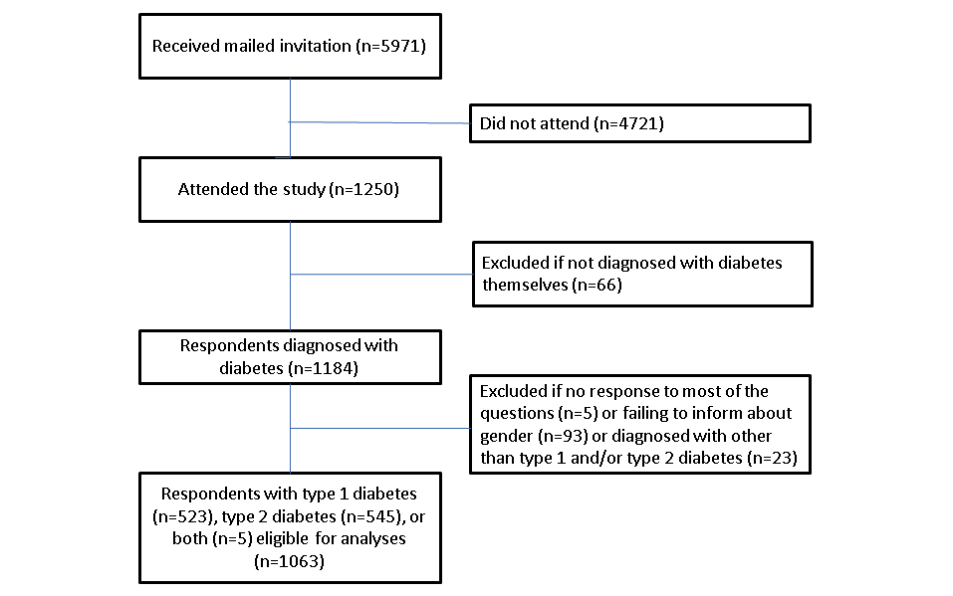
Flow chart of the study population.

It was not possible for the same respondent to fill in the questionnaire more than once. From a total of 1250 participants, we first excluded those who had not been diagnosed with diabetes themselves (n=66). This group comprised 61 family members, 4 health personnel (2 overlapping), and 3 others. We also excluded participants who failed to respond to most of the questions (n=5) and those who did not give information about gender (n=93). Finally, as we had decided to investigate T1D and T2D in this part of the study, participants with other diabetes types were excluded (n=23). The final sample included 1063 respondents (Figure 1). Of these, 523 reported having T1D, and 545 reported having T2D. A total of 5 of these were overlapping, interpreted as double diabetes [37,38].

### Variables

The 4 dependent variables were the use of apps (for mobile phone or tablet computer), search engines (such as Google), social media (such as Facebook), and video services (such as YouTube) for health purposes during the previous 12 months. For an easier interpretation of logistic regressions, and in line with previous research [34], these eHealth variables were dichotomized by merging the original 4 answering options into “never or once” and “sometimes or often.”

Education and household income were the key independent variables. Education was categorized into low (primary/part of secondary school), middle (completed secondary school), high (college/university<4 years), and highest education (college/university 4 years or more). The response options for household income were merged into 3 groups, labeled low (NOK 350,000 or less), medium (NOK 351,000-750,000), and high (NOK 751,000 or more) income. Adjustment independent variables were gender, age, and self-rated health. We grouped age in 20-year intervals. Response options for self-rated health were excellent, good, fair, bad, and very bad. The bad and very bad categories were merged because of low numbers in the very bad category (4 respondents). The 2 categories of the dichotomous response time variable were early and late respondents.

### Analyses

Data were analyzed by descriptive statistics and logistic regressions. Correlations were determined using Spearman correlation coefficients. As T1D and T2D are different diseases, and as the samples differed substantially, particularly regarding gender, age, and duration of the participants’ diabetes, we decided to stratify analyses by diabetes type. For each diagnostic category, we constructed 1 multivariable regression model for each of the dependent variables. The independent variables (gender, age, education, household income, and self-rated health) were introduced collectively into the models. We performed similar analyses after excluding the 5 participants reporting double diabetes. Owing to a relatively low response rate, we compared respondents who did not respond initially but eventually consented (late respondents) with early respondents, assuming that the late respondents were more similar to nonrespondents [39]. This was done by subsequently introducing the response time variable into the regression models. We used 95% CIs throughout the study. Stata, version 14.2 (StataCorp LLC), was used for all analyses.

### Ethics

Ethics approval was not required, which was confirmed by the Regional Committee for Medical and Health Research Ethics. This study has been approved by the Data Protection Officer (Personvernombudet) at the University Hospital of North-Norway (ref 2017/6579). The NSD resource center received no information about the participants other than their email addresses.

## Results

### Participation

In total, 1250 persons aged 18 to 89 years participated, constituting a minimum response rate of 20.9% (Figure 1). Eligible for analysis were the 523 participants with T1D and the 545 with T2D, which made a total of 1063 participants. A total of 5 of them reported having both diabetes types, and thus they entered into both groups (Figure 1) [37].

### Sample Characteristics

Mean age was 54.8 (95% CI 53.9-55.7) years; it was 47.0 (95% CI 45.7-48.3) years for those with T1D and 62.3 (95% CI 61.4-63.2) years for those with T2D. Median age was 57 years; it was 48 years (range 18-89) for T1D and 63 years (range 22-89) for T2D. Mean disease duration was 17.9 (95% CI 17.1-18.7) years; it was 23.2 (95% CI 21.9-24.6) years for T1D and 12.7 (95% CI 12.0-13.5) years for T2D, whereas median disease duration was 15 years; it was 22 years (range 0-75) for T1D and 10 years (range 0-68) for T2D.

Those who were married, working, and had good self-rated regulation of diabetes and good self-rated health made up the largest groups in both the T1D and T2D samples. For all other characteristics, the largest groups differed between the 2 diagnoses (Table 1).

Among participants with T1D, women (281/523, 53.7%), people aged 40 to 59 years (223/523, 42.6%),

people with high education (152/480, 31.7%), high household income (238/467, 51.0%), and a diabetes duration of at least 30 years (187/522, 35.8%) made up the largest groups (Table 1). In contrast, the largest groups among participants with T2D were men (342/545, 62.7%), persons aged 60 years and over (356/545, 62.7%), persons with medium education (165/510, 32.4%), medium household income (253/488, 51.9%), and persons with a diabetes duration of less than 10 years (209/542, 38.6%; Table 1). As expected, the duration of diabetes differed substantially between the 2 diagnostic groups (Table 1).

In the T1D group, 87.0% (447/514) of the participants had used 1 or more forms of eHealth sometimes or often during the previous year. The corresponding proportion for those with T2D was 77.7% (421/542). The proportion who used search engines was the largest among those with T1D, as well as those with T2D (84.0% vs 73.0%, respectively), followed by apps (55.5% vs 50.8%, respectively), social media (45.2% vs 31.2%, respectively), and video services (23.2% vs 12.3%, respectively; Table 2).

**Table 1:** Sample characteristics of participants.

Characteristic		Total sample, n (%)	Type 1 diabetes, n (%)	Type 2 diabetes, n (%)
**Gender**		**N=1063**	**n=523**	**n=545**
	Female	483 (45.43)	281 (53.7)	203 (37.3)
	Male	580 (54.57)	242 (46.3)	342 (62.7)
**Age**		**N=1063**	**n=523**	**n=545**
	18-39	192 (18.06)	178 (34.0)	14 (2.6)
	40-59	394 (37.07)	223 (42.6)	175 (32.1)
	60+	477 (44.87)	122 (23.4)	356 (65.3)
**Marital status**		**N=778**	**n=380**	**n=401**
	Single	55 (7.1)	42 (11.0)	13 (3.2)
	Married/cohabitant	723 (92.9)	338 (89.0)	388 (96.8)
**Main daily activity**		**N=984**	**n=481**	**n=507**
	Working (full-time or part-time)	519 (52.7)	308 (64.0)	215 (42.4)
	Pensioner old age	265 (26.9)	65 (13.5)	200 (39.5)
	Pensioner disability	132 (13.4)	53 (11.0)	79 (15.6)
	Pupil/student	35 (3.6)	35 (7.3)	0 (0.0)
	Other	33 (3.4)	20 (4.2)	13 (2.5)
**Education**		**N=986**	**n=480**	**n=510**
	Low (primary/part of secondary school)	107 (10.9)	39 (8.1)	68 (13.3)
	Medium (completed secondary school)	302 (30.6)	139 (29.)	165 (32.4)
	High (college/university <4 years)	300 (30.4)	152 (31.7)	150 (29.4)
	Highest (college/university, 4 years or more)	277 (28.1)	150 (31.2)	127 (24.9)
**Household income**		**N=951**	**n=467**	**n=488**
	Low (<NOK 350,000)	114 (12.0)	66 (14.1)	48 (9.8)
	Medium (NOK 351,000-750,000)	413 (43.4)	163 (34.9)	253 (51.9)
	High (NOK 751,000 or more)	424 (44.6)	238 (51.0)	187 (38.3)
**Duration of diabetes**		**N=1059**	**n=522**	**n=542**
	<10 years	333 (31.45)	127 (24.3)	209 (38.6)
	10-19 years	308 (29.08)	107 (20.5)	202 (37.3)
	20-29 years	201 (18.98)	101 (19.4)	100 (18.5)
	30 years and over	217 (20.49)	187 (35.8)	31 (5.7)
**Self-rated regulation of diabetes**		**N=1054**	**n=520**	**n=539**
	Excellent	245 (23.25)	101 (19.4)	146 (27.1)
	Good	577 (54.74)	292 (56.2)	287 (53.2)
	Fair	193 (18.31)	103 (19.8)	91 (16.9)
	Bad/very bad	39 (3.70)	24 (4.6)	15 (2.8)
**Self-rated health**		**N=1054**	**n=521**	**n=538**
	Excellent	155 (14.71)	93 (17.9)	63 (11.7)
	Good	542 (51.42)	269 (51.6)	275 (51.1)
	Fair	261 (24.76)	113 (21.7)	150 (27.9)
	Bad/very bad	96 (9.11)	46 (8.8)	50 (9.3)

**Table 2:** Proportion of participants using different kinds of electronic health (eHealth) "sometimes" or "often" during the previous 12 months.

Type of eHealth	Total sample			Type 1 diabetes^a^			Type 2 diabetes^a^		
	n/N	%	95% CI	n/N	%	95% CI	n/N	%	95% CI
One or more forms of eHealth	864/1051	82.21	79.8-84.4	*447/514*	*87.0*	*83.8-89.6*	*421/542*	*77.7*	*74.0-81.0*
Apps	556/1048	53.05	50.0-56.1	285/514	55.5	51.1-59.7	274/539	50.8	46.6-55.1
Search engines	821/1048	78.34	75.7-80.7	*431/513*	*84.0*	*80.6-86.9*	*394/540*	*73.0*	*69.0-76.6*
Social media	399/1050	38.00	35.1-41.0	*232/513*	*45.2*	*40.9-49.6*	*169/541*	*31.2*	*27.5-35.3*
Video services	183/1037	17.65	15.4-20.1	*118/506*	*23.3*	*19.8-27.2*	*66/536*	*12.3*	*9.8-15.4*

^a^Statistically significant differences between T1D and T2D are marked in italics.

### Positive Association Between Higher Education and the Use of Search Engines

We found a strong association between higher education and the use of search engines. In people with T1D, the odds were more than 3 times higher for the high education group (odds ratio, OR 3.26, 95% CI 1.34-7.96) and almost 6 times higher for the highest education group (OR 5.78, 95% CI 2.14-15.57) compared with the low education group (Table 3). Among those with T2D, the odds were more than doubled for those with high education (OR 2.17, 95% CI 1.11-4.26) and more than 3 times as high for those with the highest education (OR 3.30, 95% CI 1.58-6.89) compared with the low education group (Table 4). We found no educational differences for the use of apps, social media, or video services in any of the diagnostic categories (Tables 3 and 4).

### Type 1 Diabetes Patients With Higher Income Are Less Likely to Use Video Services

In people with T1D, we found that the middle- and high-income groups had lower odds of using video services (OR 0.51, 95% CI 0.26-0.99 and OR 0.50, 95% CI 0.26-0.98, respectively) compared with the low-income group (Table 3).

### Positive Association Between Higher Income and the Use of Apps

Among people with T1D, as well as T2D, the high income group more likely used apps, compared with the low income group (OR 3.05, 95% CI 1.63-5.71 and OR 2.06, 95% CI 1.02-4.19, respectively; Tables 3 and 4). We found no associations between household income and the use of the other eHealth types in people with T2D (Table 4).

### Gender and Age

Men with T1D and T2D were less likely to use social media (OR 0.50, 95% CI 0.34-0.74 and OR 0.62, 95% CI 0.41-0.94, respectively), and men with T2D were less likely to use search engines (OR 0.54, 95% CI 0.34-0.87), compared with women (Tables 3 and 4).

In people with T1D, higher age was inversely associated with the use of apps and search engines (Table 3). We found no statistically significant age differences in the use of eHealth among people with T2D (Table 4).

### Self-Rated Health

In many of the T1D groups that reported fair or bad/very bad health, the odds of using eHealth (except for the use of apps) were significantly higher than in the excellent health group. This was most apparent regarding the use of social media, where the odds of use were almost 3 times higher among those in bad/very bad health compared with those in excellent health (OR 2.96, 95% CI 1.34-6.54; Table 3). In the fair health group, the use of search engines was more than twice as likely as in the excellent health group (OR 2.37, 95% CI 1.03-5.46). The group with bad/very bad health was more likely to use video services (OR 2.62, 95% CI 1.15-5.97), compared with the excellent health group (Table 3). Among people with T2D reporting bad/very bad health, the odds of using search engines was significantly higher than among those in excellent health (OR 2.73, 95% CI 1.02-7.31).

There were no strong correlations (defined as rho>0.5) among the independent variables in any of the models. The strongest correlations were found for education and household income in both the T1D and T2D models (rho 0.2757 and 0.2555, respectively). Performing the regression analyses after excluding the 5 participants with “double diabetes” did not alter the results.

**Table 3:** Associations of using electronic health "sometimes" or "often" in people with type 1 diabetes.

Characteristics		Apps (n=467), OR^a^ (95% CI)^b^	Search engines (n=466), OR (95% CI)^b^	Social media (n=466), OR (95% CI)^b^	Video services (n=463), OR (95% CI)^b^
**Gender**					
	Female^c^	1.00	1.00	1.00	1.00
	Male	1.40 (0.95-2.06)	0.71 (0.41-1.21)	*0.50 (0.34-0.74)*	1.43 (0.91-2.24)
**Age**					
	18-39 years^c^	1.00	1.00	1.00	1.00
	40-59 years	*0.52 (0.33-0.84)*	*0.34 (0.16-0.74)*	1.20 (0.76-1.89)	0.84 (0.50-1.40)
	60+ years	*0.45 (0.26-0.77)*	*0.23 (0.10-0.52)*	0.72 (0.42-1.23)	0.59 (0.31-1.12)
**Education**					
	Low education^c^ (primary/part of secondary school)	1.00	1.00	1.00	1.00
	Medium education (completed secondary school)	0.84 (0.39-1.80)	1.64 (0.70-3.82)	1.03 (0.48-2.20)	0.89 (0.36-2.16)
	High education (college/university, <4 years)	0.81 (0.38-1.73)	*3.26 (1.34-7.96)*	0.95 (0.44-2.03)	1.10 (0.45-2.68)
	Highest education (college/university, 4 years or more)	0.84 (0.39-1.82)	*5.78 (2.14-15.57)*	0.79 (0.37-1.71)	0.97 (0.39-2.40)
**Household income**					
	Low income^c^ (NOK <350,000)	1.00	1.00	1.00	1.00
	Medium income (NOK 351,000-750,000)	1.26 (0.68-2.33)	1.11 (0.49-2.50)	1.00 (0.54-1.86)	*0.51 (0.26-0.99)*
	High income (NOK 751,000 or more)	*3.05 (1.63-5.71)*	1.60 (0.68-3.78)	1.55 (0.83-2.89)	*0.50 (0.26-0.98)*
**Self-rated health**					
	Excellent health^c^	1.00	1.00	1.00	1.00
	Good health	1.31 (0.78-2.20)	1.88 (0.95-3.71)	*1.99 (1.15-3.45)*	0.96 (0.52-1.78)
	Fair health	1.68 (0.91-3.12)	*2.37 (1.03-5.46)*	*2.46 (1.30-4.64)*	1.08 (0.53-2.23)
	Bad/very bad health	1.96 (0.89-4.32)	2.13 (0.69-6.60)	*2.96 (1.34-6.54)*	*2.62 (1.15-5.97)*

^a^OR: odds ratio.

^b^Statistically significant findings are marked in italics.

^c^Reference groups.

**Table 4:** Associations of using electronic health "sometimes" or "often" in people with type 2 diabetes.

Characteristics		Apps (n=478), OR^a^ (95% CI)^b^	Search engines (n=479), OR (95% CI)^b^	Social media (n=480), OR (95% CI)^b^	Video services (n=476), OR (95% CI)^b^
**Gender**					
	Female^c^	1.00	1.00	1.00	1.00
	Male	0.92 (0.63-1.36)	*0.54 (0.34-0.87)*	*0.62 (0.41-0.94)*	0.72 (0.40-1.28)
**Age**					
	18-39 years^c^	1.00	1.00	1.00	1.00
	40-59 years	0.83 (0.23-2.96)	1.17 (0.21-6.37)	1.11 (0.30-4.12)	0.88 (0.16-4.70)
	60+ years	0.83 (0.24-2.91)	0.86 (0.16-4.54)	0.77 (0.21-2.82)	0.58 (0.11-3.06)
**Education**					
	Low education^c^ (primary/part of secondary school)	1.00	1.00	1.00	1.00
	Medium education (completed secondary school)	1.29 (0.71-2.35)	1.74 (0.92-3.29)	1.06 (0.56-2.04)	0.69 (0.27-1.74)
	High education (college/university, <4 years)	1.42 (0.76-2.63)	*2.17 (1.11-4.26)*	1.05 (0.54-2.05)	1.18 (0.48-2.91)
	Highest education (college/university, 4 years or more)	1.28 (0.68-2.43)	*3.30 (1.58-6.89)*	0.84 (0.42-1.69)	0.91 (0.35-2.38)
**Household income**					
	Low income^c^ (NOK <350,000)	1.00	1.00	1.00	1.00
	Medium income (NOK 351,000-750,000)	1.66 (0.84-3.26)	1.11 (0.52-2.33)	1.20 (0.58-2.50)	1.13 (0.39-3.21)
	High income (NOK 751,000 or more)	*2.06 (1.02-4.19)*	2.25 (0.99-5.11)	1.15 (0.53-2.46)	0.92 (0.31-2.78)
**Self-rated health**					
	Excellent health^c^	1	1	1	1
	Good health	1.11 (0.62-1.97)	1.42 (0.74-2.74)	1.08 (0.57-2.05)	1.11 (0.43-2.89)
	Fair health	1.20 (0.64-2.23)	1.56 (0.77-3.19)	1.37 (0.69-2.70)	1.75 (0.66-4.64)
	Bad/very bad health	1.44 (0.66-3.18)	*2.73 (1.02-7.31)*	1.06 (0.45-2.51)	1.13 (0.31-4.06)

^a^OR: odds ratio.

^b^Statistically significant findings are marked in italics.

^c^Reference groups.

## Discussion

### Principal Findings

We found that 87.0% (447/514) of people with T1D and 77.7% (421/542) of people with T2D had used 1 or more forms of eHealth sometimes or often during the previous year. In both diagnostic groups, the proportion using search engines was the largest, followed by apps, social media, and video services. Those with higher levels of education had higher odds of using search engines, whereas we found no educational differences for the use of apps, social media, or video services. In both diagnostic groups, those with high income more likely used apps, whereas T1D patients with medium and high income had lower odds of using video services. Men in both diagnostic groups used social media less than women did. In people with T1D, higher age was inversely associated with the use of apps and search engines, whereas those in poorer health had higher odds of using eHealth, particularly social media. There was no association between self-rated health and the use of apps. Among people with T2D, those with bad/very bad health had significantly higher odds of using search engines compared with those in excellent health.

### Overall Use of Electronic Health Among People With Diabetes

We revealed a high overall use of eHealth, and search engines were the most commonly used platforms. This conforms with the study by Hong et al, which reported that seeking health information on the Web has been the most typical health-related internet use in the general US population of older adults (55 years and over), increasing from 57% in 2003 to 80% in 2011 [9]. Considering the high mean age in this study’s sample, this study’s rates for a Norwegian diabetes population can be seen as a confirmation of this trend, and for T1D, they can be seen as an extension of it. This is also in line with previous research, indicating that long-term illness and good health, which characterize this study’s sample, are related to increased use of eHealth [22]. The high overall use of eHealth suggests increased efforts in providing high-quality electronic information and services tailored for people with diabetes.

### Differences According to Education

There was a strong association between higher education and the use of search engines in both diagnostic groups. This is in line with most other studies of education and Web-based health information seeking in general and disease-specific populations [10,25,40]. The finding might be explained by higher-educated groups’ capabilities and experiences of seeking out, finding, understanding, and making sense of health- and disease-related information [25,40]. It might also reflect educational differences in engagement with health, health care systems, and health care activities [25]. This might be reinforced by information tailored for people with higher education more than for people with lower education, hindering future searches among those who experienced that they did not fully understand what was found [25]. Notably, education was not significantly associated with the use of apps, social media, or video services in this study. Kontos et al even found lower levels of education to be associated with increased use of social media for health purposes [25], which might indicate that social media information is experienced as more accessible and useful in groups with lower education. The peer-to-peer interactions and social and emotional support provided by

social media may be of significance in this regard [41]. Regarding the use of apps, our finding of no association with education supports other recently conducted studies [42,43]. This might indicate benefit from communication through apps, social media, and video services, regardless of education, whereas higher-educated people might additionally experience benefit from information through literature and other texts, whether internet-based or not. When targeting people with lower education, one might thus consider providing information through apps, videos, or social media. This is important, as it might have consequences for engagement in healthy lifestyle behavior and the ability to achieve better health outcomes.

### Differences According to Household Income

Health apps may be used for a wide variety of health purposes, including self-management and control of diabetes. In both diagnostic categories, the high-income group had higher odds of using apps than the low income group. The association was stronger for T1D than for T2D (Tables 3 and 4). In contrast, an Australian study recently reported no association between SES and the use of apps for T1D and T2D [42]. This is interesting even if findings are not directly comparable. A possible explanation of our finding might be the costs of downloading some of the apps. As the use of apps is still increasing, another possible explanation might be that novel solutions and treatments reach people in higher socioeconomic groups first [19-21]. We found that T1D patients with medium and high income had lower odds of using video services compared with the low-income group. This might indicate that people with low income are more likely to benefit from video-based information regarding health issues. However, this association was not found for T2D. A possible explanation might be the higher age in this group and less use of video services compared with the T1D group (Tables 1 and 2). In a general US population, higher household income was significantly associated with seeking health information on the Web in 2003, 2005, and 2011-12 [9]. This is only partly supported by this study’s results, as we did not find any significant association between household income and the use of search engines or social media.

### Differences According to Gender

The only significant gender differences in this study were that men in both diagnostic categories used social media less than women and that men with T2D used search engines less than women. Previous research reports a general trend that women have a higher engagement in health issues, eHealth, and social media, and women often act as a liaison for their family [9,25], which might underpin and explain these findings. Nevertheless, we found no gender differences for the use of apps and video services. Previous research has produced conflicting results regarding gender and the use of apps [43,44], and evidence is scarce about predictors for the use of video services. The small gender differences in this study (Tables 3 and 4) are in line with the trend reported by Hong et al that the digital health divide between genders narrowed from 2003 and was no longer significant in 2011 [9].

### Differences According to Age

Higher age was inversely associated with the use of apps and search engines in people with T1D. Others have specifically described an association between younger age and app use among people with diabetes [42]. In line with these results, previous research consistently reports that older people use eHealth less than younger people, both in general populations and elderly populations, as well as in populations with chronic disease [10,11,22,25]. Another possible explanation is that people with T1D are likely to have a longer disease duration the older they are, as incidence of T1D decreases with increasing age. With their greater experience in living with diabetes, they may not use apps or search engines as much as if they were more recently diagnosed. This is consistent with the duration of diabetes described in Table 2. In addition, the lower use of apps and search engines among older people with T1D might partly be because of less use of mobile devices in general, as well as lower education in older age groups [13,45]. Age-sensitive information design for elderly people could be an area for development. However, in the T2D group, we found no age-related differences in the use of eHealth. The association regarding app and search engine use described above for the T1D group may be consistent with the lack of association with age among those with T2D. As T2D incidence is higher with increasing age, more of the T2D sample is at any given age likely to be more recently diagnosed than the veterans of T1D at their comparable ages, thus explaining that eHealth use does not differ by age in the T2D group. Hong et al described a narrowing of the age divide in the general US population from 2003 to 2011 [9]. This study’s findings suggest a disappearance of the age divide in the T2D group, as well as regarding the use of social media and video services in the T1D group. Our findings are not surprising, as the use of eHealth is increasing rapidly among elderly people in the western world [10,13,46], and the elderly population is gradually transforming from “digital immigrants” (having to learn and acquire digital activity as adults) to “digital natives” (having grown up with digital technology) [25]. Thus, the inverse association between age and eHealth use reported in previous research might not be sustained in the future [13,46] .

### Differences According to Self-Rated Health

Previous research has produced conflicting results regarding the relationship between health status and the use of eHealth [47]. A striking finding in this study was that those reporting bad/very bad health were almost 3 times as likely to use social media compared with those in excellent health. Access of shared information through social media might give valuable fellowship, along with clinical and emotional support for people with T1D, particularly in periods of poorer health [41]. In people with T2D, we found a positive association between bad/very bad health and the use of search engines, which is in line with the illness behavior model, stating that people in poorer health more likely seek disease-related information on the Web [23]. We found no significant associations between self-rated health and app use (Tables 3 and 4). Others have found an association in terms of better outcomes of health parameters among app users, stating that the use of apps contributes to better disease management and health outcomes [42].

### Limitations and Strengths

Limitations and strengths have been discussed in detail in our first study in this project [11]. One limitation is the low estimated participation rate. However, response rate must not be confused with response quality [39]. Older people dominated among the late respondents compared with the early respondents [11]. As late respondents might be more similar to nonrespondents [39], seniors might be underrepresented in this study.

Distribution of the questionnaire by email is another limitation, which excluded those who do not use the internet. As 98% of Norwegian households have internet access, we do not think this affected our results significantly [11]. It is well known that women, healthier persons, higher socioeconomic groups, and middle-aged people are more likely to participate in surveys [11]. This suggests that women, people around 40 to 80 years, people in better health, and higher socioeconomic groups might be overrepresented in this study. As different factors might pull the tendency in different directions or level each other out, it is not possible to judge the magnitude or direction of a possible nonresponse bias. The low response rate is in itself not an indication of low representativeness [48]. We suggest that nonresponse bias posed a limited threat to this study’s validity; however, generalization must be made with caution. We investigated socioeconomic differences in the form of self-reported education and self-reported household income. Education was measured at the individual level, whereas household income was measured at family level, which is a limitation. The answers regarding household income might be less accurate than those regarding one’s own education. We thus consider education a more solid measure than household income. However, we think that both these aspects of SES, despite limitations, offer a broader picture than one of them might do alone.

Recall bias might have occurred for all aspects of this questionnaire study. Other relevant limitations explored in the first study were the validity of self-reported data, the cross-sectional study design, and interest in the subject studied [11]. Finally, we cannot exclude the possibility of unmeasured confounders of the reported associations. This study also has some strengths, which are similar to the strengths discussed in the first paper in this series [11]. The most important strength is the focus on a scarcely investigated research field. Other strengths are the detailed questionnaire specifically tailored to people with diabetes, the recruitment of participants from all of Norway, the inclusion of a wide age span of participants, and the opportunity to analyze the data shortly after they were collected. Finally, the collection of data in cooperation with the NDA enabled us to develop an excellent user participation, with a large and important group of health care users.

### Conclusions

Overall, this study indicates a digital divide among people with diabetes in Norway, with consequences that may contribute to shaping inequalities in health outcomes. We want to highlight the strong relationship between higher education and the use of search engines, along with the finding that educational level was not associated with differences in the use of apps, social media, and video services. We also revealed that the use of video services was more likely in lower income groups. Collectively, our findings suggest that information through apps, social media, and video services might be a good choice when targeting lower educational groups. In society’s effort to reduce inequalities in health outcomes, clinicians and health care leaders should be aware of these inequalities in eHealth use to design adequate health communication strategies for different target groups, particularly according to educational level. More research is needed to confirm our findings.

## Appendix

Multimedia Appendix 1 Survey questionnaire.

## Abbreviations

eHealth: electronic health
NDA: Norwegian Diabetes Association
NSD: Norwegian Centre for Research Data
OR: odds ratio
SES: socioeconomic status
T1D: type 1 diabetes
T2D: type 2 diabetes
