# Supplementary material for: Inequalities in the Use of eHealth Between Socioeconomic Groups Among Patients With Type 1 and Type 2 Diabetes: Cross-Sectional Study
Source: J Med Internet Res. 2019 May 29;21(5):e13615. doi: 10.2196/13615 (PMC6658320; doi:10.2196/13615)
Supplement: Multimedia Appendix 1 [file jmir_v21i5e13615_app1.pdf]

Questionnaire DIAcare

BACKGROUND INFORMATION

1. Do you yourself have diabetes diagnosed by a doctor? (yes/no)

2. If no, what is the reason that you are a member of the Norwegian Diabetes Association? (check one or more boxes)
    a. Health professional
    b. Family member
    c. Other…………………….

Those who have chosen the response alternative "No" to the question "Do you have diabetes diagnosed by a doctor?" can end the survey here.

Those with the response alternative "Yes" to the question "Do you have diabetes diagnosed by a doctor?" have access to the rest of the survey.

3. What is your gender (woman/man)?

4. What is your age? (number of years)

YOUR OWN HEALTH

5. What type of diabetes do you have? (check one box) (type 1/type 2/gestational diabetes/other type)

6. How many years have you had diabetes? (number of years)

7. How is your diabetes being treated? (check one or more boxes) (insulin/tablets/diet and lifestyle)

8. How would you say that your diabetes is controlled? (check one box) (very well/well/neither well nor poorly/poorly/very poorly)

9. How do you rate your own health in general? (check one box) (very good/good/neither good nor poor/ poor/very poor)

10. Have you ever smoked or do you smoke every day? (check one box) (never/yes, now/yes, previously)

11. Do you have or have you had high blood pressure? (no/yes, previously but not now/yes, now)

12. Have you had a heart attack (myocardial infarction)? (no/yes)

13. Do you have or have you had heart failure? (no/yes, previously but not now/yes, now)

14. Do you have or have you had angina pectoris/chest pain due to heart disease? (no/yes, previously but not now/yes, now)

15. Have you had a stroke/brain haemorrhage? (no/yes, previously but not now/yes, now)

16. Do you have or have you had kidney disease? (no/yes, previously but not now/yes, now)

17. Do you have or have you had cancer? (no/yes, previously but not now/yes, now)

18. Do you have or have you had mental illness? (no/yes, previously but not now/yes, now)

19. How would you describe your health today (check one box for each of the five areas)

    a. Walking
        i. I have no problems in walking around
        ii. I have slight problems in walking around
        iii. I have moderate problems in walking around
        iv. I have severe problems in walking around
        v. I am unable to walk around

    b. Personal care
        i. I have no problems washing or dressing myself
        ii. I have slight problems washing or dressing myself
        iii. I have moderate problems washing or dressing myself
        iv. I have severe problems washing or dressing myself
        v. I am unable to wash or dress myself

    c. Common tasks
        i. I have no problems doing my usual activities
        ii. I have slight problems doing my usual activities
        iii. I have moderate problems doing my usual activities
        iv. I have severe problems doing my usual activities
        v. I am unable to do my usual activities

    d. Pain/discomfort
        i. I have neither pain nor discomfort
        ii. I have slight pain or discomfort
        iii. I have moderate pain or discomfort
        iv. I have severe pain or discomfort
        v. I have extreme pain or discomfort

    e. Anxiety/depression
        i. I am neither anxious nor depressed
        ii. I am slightly anxious or depressed
        iii. I am moderately anxious or depressed
        iv. I am severely anxious or depressed
        v. I am extremely anxious or depressed

USE OF THE INTERNET/E-HEALTH

20. During the past 12 months, have you used the Internet for information about health and illness? (check one box for each of the four categories)
    a. Apps for smartphone or tablet computer (never/once/sometimes/often)
    b. Search engines (like Google) (never/once/sometimes/often)
    c. Social media (like Facebook) (never/once/sometimes/often)
    d. Video services (like YouTube) (never/once/sometimes/often)

21. During the past 12 months, have you used apps for a smartphone or tablet computer for follow-up of your own diabetes (check one box) (never/less than once a month/once a month/once a week/every day)

22. During the past 12 months, have you used one or more of the following portals for information about health and illness? (check one box for each of the eight categories)
    a. diabetes.no (never/once/sometimes/often)
    b. levmeddiabetes.no (never/once/sometimes/often)
    c. helsebiblioteket.no (never/once/sometimes/often)
    d. helsenorge.no (never/once/sometimes/often)
    e. lommelegen.no (never/once/sometimes/often)
    f. nhi.no (never/once/sometimes/often)
    g. apotek1.no (never/once/sometimes/often)
    h. other portals (never/once/sometimes/often)

23. How do you rate the quality of the information in these health portals? (check one box for each of the eight categories)
    a. diabetes.no (very good/good/neither good nor bad/ bad/very bad/not considered)
    b. levmeddiabetes.no (very good/good/neither good nor bad/ bad/very bad/not considered)
    c. helsebiblioteket.no (very good/good/neither good nor bad/ bad/very bad/not considered)
    d. helsenorge.no (very good/good/neither good nor bad/ bad/very bad/not considered)
    e. lommelegen.no (very good/good/neither good nor bad/ bad/very bad/not considered)
    f. nhi.no (very good/good/neither good nor bad/ bad/very bad/not considered)
    g. apotek1.no (very good/good/neither good nor bad/ bad/very bad/not considered)
    h. other portals (very good/good/neither good nor bad/ bad/very bad/not considered)

24. Based on the information you have found on the Internet, have you (check one box for each of the four categories)
    a. Decided to consult a doctor, when you would otherwise NOT have consulted one? (never/once/sometimes/often)
    b. Decided NOT to consult a doctor, when you would otherwise have consulted one? (never/once/sometimes/often)
    c. Discussed the information with a doctor? (never/once/sometimes/often)
    d. Changed your medicine without talking to a doctor? (never/once/sometimes/often)
    e. Become unsure about whether you have received the right diagnosis? (never/once/sometimes/often)
    f. Become unsure about whether you have received the right treatment? (never/once/sometimes/often)

g. Changed your lifestyle? (never/once/sometimes/often)
h. Felt anxious? (never/once/sometimes/often)
i. Felt reassured? (never/once/sometimes/often)
j. Felt better informed? (never/once/sometimes/often)
k. Felt more confused? (never/once/sometimes/often)

25. During the past 12 months, have you searched for health information on the Internet in order to (check one or more boxes)
    a. Decide whether you should go to a doctor?
    b. Prepare for a visit to the doctor?
    c. Obtain information after a visit to the doctor?

26. During the past 12 months, have you taken part in any online group for people with diabetes? (yes/no)

27. Have you communicated over the Internet with your primary care doctor/primary care doctor's office during the past 12 months? (yes/no, number of times)

28. If yes, have you used Internet communication to (check one or more boxes)
    a. Book an appointment (yes/no)
    b. Communicate about your health/illness (yes/no)
    c. Renew a prescription (yes/no)
    d. Other purpose (yes/no)

USE OF HEALTH SERVICES

29. During the past 12 months, because of your own health, have you consulted a primary care doctor/general practitioner (GP) (your own or a substitute) (Yes/no/number of times)

30. If yes, how many of these consultations were with your own primary care doctor (not with a substitute doctor)?

31. During the past 12 months, because of your own health, have you consulted a doctor on duty outside working hours? (Yes/no/number of times)

32. During the past 12 months, because of your own health, have you consulted a psychiatrist/psychologist outside hospitals? (Yes/no/number of times)

33. During the past 12 months, because of your own health, have you consulted a medical specialist outside hospitals (apart from your primary care doctor/a general practitioner (GP)/a psychiatrist)? (Yes/no/number of times)

34. During the past 12 months, because of your own health, have you consulted a psychiatric hospital outpatient clinic? (Yes/no/number of times)

35. During the past 12 months, because of your own health, have you consulted another hospital outpatient clinic (apart from a psychiatric outpatient clinic)? (Yes/no/number of times)

36. During the past 12 months, have you been admitted to hospital? (No, have not been admitted to hospital/Yes, admitted to a psychiatric hospital/ Yes, admitted to other (somatic) hospital)

37. If "yes, admitted to psychiatric hospital", how many times have you been admitted? (number of times)

38. If "yes, admitted to other (somatic) hospital", how many times have you been admitted? (number of times)

39. Do you have health insurance that gives you access to private health services? (yes/no/do not know)

40. How long have you had your current primary care doctor (regular GP)? (check one box) (less than 1 year/1-2 years/3-4 years/ more than 4 years)

MORE BACKGROUND INFORMATION

41. In which region are you currently living? (check one box) (Nordland, Troms, Finnmark, Svalbard/ Trøndelag, Møre og Romsdal/ Rogaland, Hordaland, Sogn og Fjordane/ Agder, Telemark, Vestfold, Buskerud, Østfold, Akershus, Oslo, Hedmark, Oppland)

42. What/where is your country of birth? (check one box) (Norway/ Europe outside Norway/ Pakistan/Asia outside Pakistan/ Africa/ North America/ South America/ Australia or New Zealand)

43. What is your highest completed education? (check one box)
    a. primary and lower secondary school/secondary modern school (framhaldsskole)/college (folkehøyskole) up to 10 years
    b. Vocational training/high school (realskole) /upper secondary school/sixth form of comprehensive school (gymnas) minimum 3 years
    c. University college/university, less than 4 years
    d. University college/university, 4 years or more

44. Do you live alone or with other people? (alone/with others, if yes how many)

45. Do you live with your spouse or cohabitant?

46. Do you live with parents/guardians? (if yes, number)

47. What was your household's total gross income last year? |(check one box) Include all income from work, social security, social assistance and similar (under NOK 150000 /150000-350000/351000-550000/55100-750000/751000-1000000/over 1000000)

48. What is your main daily activity? (check one box)
    e. Employed full-time
    f. Employed part-time
    g. Homemaker

h. Retirement pensioner
i. Disability pensioner/receive a work assessment allowance (arbeidsavklaringspenger)
j. Receive social assistance benefits (sosialstønad).
k. Unemployed
l. School pupil/student
m. Military
n. Other
